# Apicomplexan parasites in urban corvids: Presence of *Toxoplasma gondii* DNA and first detection of *Lankesterella* and *Isospora* DNA in the brain of Eurasian magpies, *Pica pica*

**DOI:** 10.1016/j.crpvbd.2026.100417

**Published:** 2026-07-22

**Authors:** Barbara Tuska-Szalay, Klaudia Gaál, Martin Horváth, Nóra Takács, Gergő Keve, Lajos Szabó, Imre Gergely, Sándor Hornok

**Affiliations:** a Department of Parasitology and Zoology, University of Veterinary Medicine, Budapest, Hungary; b Hársfa Street Small Animal Clinic, Pécs, Hungary; c BUD Airport Ltd. Bird and Wildlife Control Team, Budapest, Hungary; d Dömsöd Agricultural Investment Ltd., Dömsöd, Hungary; e HUN-REN-UVMB Climate Change: New Blood-sucking Parasites and Vector-borne Pathogens Research Group, Budapest, Hungary

**Keywords:** Cystogenic coccidia, Corvidae, *Pica pica*, Urbanization, 18S rRNA

## Abstract

Corvids, including crows, magpies, ravens and jays, are among the most abundant and adaptable bird species in urban and peri-urban environments, where their proximity to humans and domestic animals may facilitate the circulation of parasites. This study aimed at analyzing the presence of DNA of *Toxoplasma gondii*, *Neospora caninum* and other coccidia with neurotropism in corvids. A total of 279 brain samples collected at two peri-urban locations in Hungary were examined using molecular methods. The majority of sampled individuals were Eurasian magpies (*Pica pica*; *n* = 273). In addition, Eurasian jays (*Garrulus glandarius*; *n* = 4) and hooded crows (*Corvus cornix*; *n* = 2) were included. Apicomplexan DNA was only detected in the brain of *P. pica*. In this species, *T. gondii* was identified with a prevalence of 3.7%, for the first time in *P. pica* in Hungary and Central Europe. Furthermore, DNA of a *Lankesterella* sp. was detected in 4% of the specimens of *P. pica* examined, representing the first molecular evidence of its presence in any corvid species. Because the 18S rRNA sequence of this *Lankesterella* sp. showed at least 1.3% sequence difference from already reported isolates/species but was 100% identical among all 11 infected *P. pica* at two locations, this isolate may represent a host-associated genotype. DNA of both *T. gondii* and *Lankesterella* sp. was detected in three birds. In the brain of one *P. pica*, an *Isospora* species was detected, showing the closest sequence identity to *Isospora phylidonyrisae* and *Isospora coronoideae*, which have so far been reported from avian hosts in Australia. These findings broaden our knowledge on the diversity of apicomplexan parasites harbored by *P. pica* and provide a basis for future studies assessing the potential One Health significance of corvids in urban environments.

## Highlights

• Apicomplexan DNA was detected in the brain of 7% of Eurasian magpies (*Pica pica*).
• First report of *Lankesterella* infection in a corvid species.
• First detection of *Toxoplasma gondii* in magpies in Hungary.
• Detected *Isospora* species are closely related to Australian avian isolates.
• Corvid-associated apicomplexan parasites may represent a relevant focus for future One Health studies.

## Graphical abstract

**Figure:**
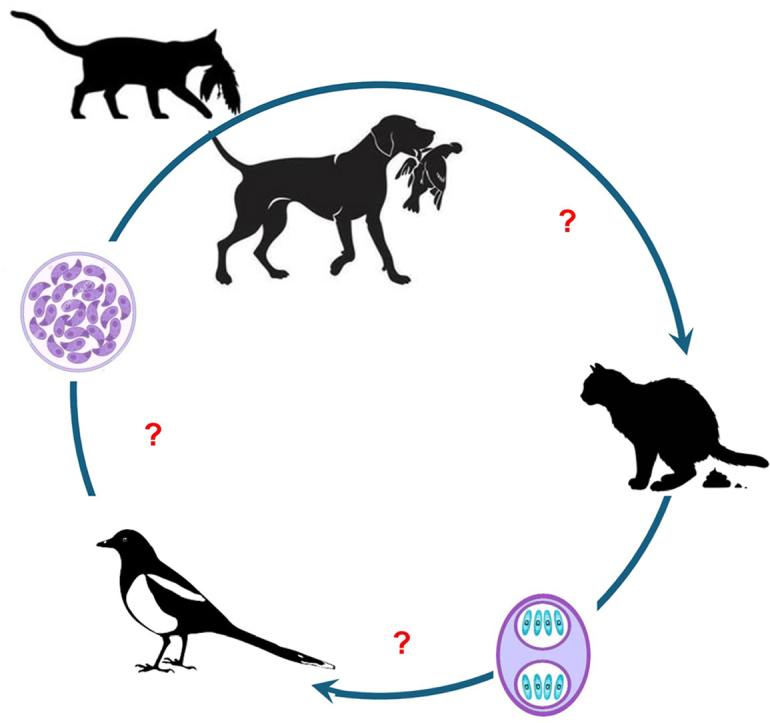

## 1 Introduction

Members of the family Corvidae (order Passeriformes), including crows, magpies, ravens and jays, occur worldwide. Many of these species inhabit a broad range of urban environments, from the outskirts to centers of cities (Benmazouz et al., 2021). Corvids are frequently exposed to environmental protozoan parasites through their omnivorous habit and opportunistic feeding on plants, animals, and human-derived food. In these avian hosts, the most commonly detected protozoans are *Toxoplasma gondii*, *Sarcocystis* spp., and *Leukocytozoon* spp., while less frequently they may harbor *Plasmodium* spp. (Freund et al., 2016; Shurulinkov et al., 2018; Juozaitytė-Ngugu et al., 2021, 2022; Gherman et al., 2025). Further protozoans from phylum Apicomplexa that may cross the blood-brain barrier include species which are seldom reported or have never been detected from corvids, such as *Neospora caninum*, *Lankesterella* and *Isospora* spp. (Abdoli et al., 2018; Juozaitytė-Ngugu et al., 2021; Parsa et al., 2023).

Considering cystogenic coccidia (Apicomplexa: Sarcocystidae), *T. gondii* is of high public health concern. Although felids represent the only definitive hosts able to shed environmentally resistant oocysts, a broad spectrum of warm-blooded animals serve as intermediate hosts, including birds that become infected through the ingestion of sporulated oocysts. Although avian toxoplasmosis is primarily considered in an epidemiological context, fatal infections have been documented in several bird species, including corvids (Dubey, 2002; Hill and Dubey, 2002; Dubey et al., 2020). In Europe, data regarding the prevalence of *T. gondii* in corvid species remain limited. Nonetheless, infections have been reported in Spain, Italy and Romania in Eurasian jays (*Garrulus glandarius*), hooded crows (*Corvus cornix*), Eurasian magpies (*Pica pica*), rooks (*Corvus frugilegus*), jackdaws (*Coleus monedula*) and common ravens (*Corvus corax*) (Darwich et al., 2012; Molina-López et al., 2012; Mancianti et al., 2020; Gherman et al., 2025).

*Neospora caninum* (Sarcocystidae) can form tissue cysts in the brain of various avian species (Darwich et al., 2012; Abdoli et al., 2018; Liu et al., 2019b). Corvids usually become infected through the ingestion of oocysts from habitats contaminated by the definitive host or by consuming tissue cysts present in intermediate hosts. Thus, corvids may act as potential sources of infection with *N. caninum* to dogs (Dubey et al., 2017; Abdoli et al., 2018). The presence of *N. caninum* has been reported in *C. cornix* and *C. monedula* in Israel and Iran (Salant et al., 2015; Abdoli et al., 2018), while in Europe it has been detected only in *P. pica* in Spain (Darwich et al., 2012).

Species of the genus *Sarcocystis* (Sarcocystidae) are characterized by an obligate two-host prey-predator life cycle. Birds serve as definitive hosts for at least 17 species of *Sarcocystis* worldwide (Dubey et al., 2015; Juozaitytė-Ngugu et al., 2021). To date, apart from *Sarcocystis ovalis*, which has been conclusively linked to corvids as definitive hosts (Gjerde and Dahlgren, 2010; Irie et al., 2017), additional *Sarcocystis* species have been identified in the intestinal contents, muscle tissues and brain of corvids (Dubey et al., 2015; Maier et al., 2015; Juozaitytė-Ngugu et al., 2021).

Species of *Lankesterella* (Lankesterellidae) were initially known to infect amphibians and reptiles; their presence has recently been confirmed in birds (Chagas et al., 2021; Keckeisen et al., 2024; Saravana Bhavan Venkatachalam et al., 2025). Transmission between vertebrate hosts, as observed in *Hepatozoon* spp., occurs *via* ingestion of infected vectors, primarily mites, ticks and leeches (Desser, 1993; Poinar, 2009). Once in the bloodstream, sporozoites can disseminate to various organs, including the brain, as evidenced by their detection in the brain tissue of several passerine species, e.g. barn swallow (*Hirundo rustica*), white wagtail (*Motacilla alba*), European robin (*Erithacus rubecula*), and common whitethroat (*Curruca communis*). In contrast, the occurrence of any *Lankesterella* spp. in the brain tissue of corvids has not been reported (Keckeisen et al., 2024).

Passerine birds may also become infected with *Isospora* spp. (Eimeriidae) through the ingestion of oocysts from the environment. Some species of this genus cause enteric isosporosis while others exhibit prolonged development extraintestinally, resulting in systemic isosporosis involving the spleen, liver, heart, lungs, and brain (Adkesson et al., 2005; Schrenzel et al., 2005; Flach et al., 2022; Keckeisen et al., 2024). As a result, systemic isosporosis is an important cause of mortality in passerine birds, particularly in juveniles and captive individuals (Rossi et al., 1997; Amaral et al., 2025). Of particular interest, cerebral localization has thus far been documented exclusively in the painted bunting (*Passerina ciris*), Java sparrow (*Padda oryzivora*), and Eurasian tree sparrow (*Passer montanus*) (Keckeisen et al., 2024).

Corvids are common inhabitants of urban and peri-urban environments and may facilitate the circulation of pathogens between wildlife, domestic animals and humans. However, relatively little is known about the diversity and tissue distribution of apicomplexan parasites in European corvid populations. Therefore, the aim of the present study was threefold. First, to elucidate which apicomplexan parasites infect the brain tissue of corvids, as neural tissue represents a suitable matrix for molecular detection due to the ability of several apicomplexan parasites to disseminate to and persist within the central nervous system. Secondly, to characterize the taxonomic relationships of these protozoans with molecular phylogenetic methods. Thirdly, to interpret the results according to the epidemiological implications that the detected apicomplexans might have for other avian hosts or companion animals kept outdoors.

## 2 Materials and methods

### 2.1 Sample collection

**Supplementary Fig. S1:**
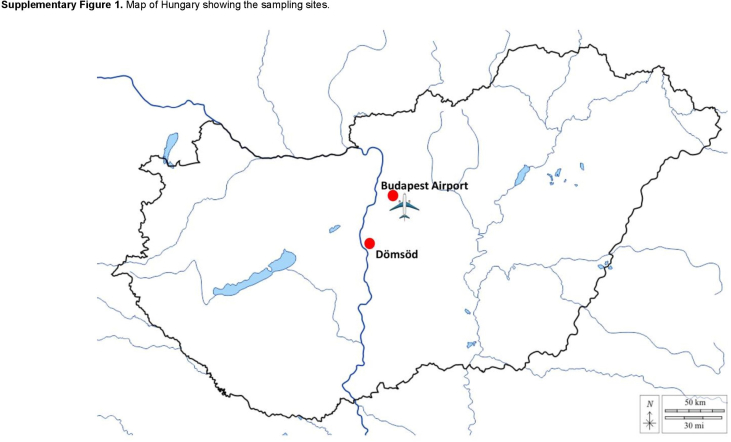
Map of Hungary showing the sampling sites.

In this study, a total of 279 corvids were included from two peri-urban locations in Hungary: the Budapest Liszt Ferenc International Airport (BUD; *n* = 62) and the vicinity of Dömsöd in Pest County (*n* = 217) (Supplementary Fig. S1). Both areas are situated under dry continental climate and can be characterized by surroundings of sparse tree covering (mainly locusts, *Robinia* sp.) and gardens where pet animals (mainly dogs) are kept. These birds were culled between 2021 and 2022 for population control purposes in accordance with national regulations (Decree No. 79/2004 (V.4.) of the Ministry of Agriculture and Rural Development on the implementation of Act LV of 1996 on the protection of game, game management, and hunting). The majority of sampled individuals were Eurasian magpies (*Pica pica*; *n* = 273); additionally, Eurasian jays (*Garrulus glandarius*; *n* = 4; all from Dömsöd) and hooded crows (*Corvus cornix*; *n* = 2; all from BUD) were included. At the time of culling, no clinical abnormalities were noted on the birds, nor were any pathological lesions observed during autopsy. The age, sex, and body condition of the examined individuals could not be assessed at the time of sampling.

During necropsy, the skull was opened and the meninges were ruptured, after which brain tissue samples were collected from the central region of the brain using sterile dissecting instruments. Samples were placed into sterile Sarstedt tubes and stored at −20 °C until further processing.

### 2.2 Molecular and phylogenetic analyses

DNA was extracted individually from approximately 30 mg of brain tissue collected from the dorsal portion of the cerebral hemispheres using the QIAamp DNA Mini Kit (Qiagen, Hilden, Germany) according to the manufacturer’s tissue protocol. An extraction control was included to monitor potential cross-contamination, and the resulting DNA samples were subsequently labelled PB1-PB279. To minimize the risk of contamination, all stages of the molecular workflow, including DNA extraction, PCR preparation, amplification, and post-PCR processing, were conducted in physically separate rooms. Furthermore, no-template controls were included in every primary and nested PCR run and processed alongside the samples to monitor for potential contamination throughout the test.

DNA samples were first analyzed for the presence of certain groups of apicomplexan parasites by a general screening assay, followed by PCRs amplifying longer parts of the 18S rRNA gene for genotyping with sequence comparisons and phylogenetic analyses. The primers used in these assays are listed in Table 1 (Ho et al., 1996; Chagas et al., 2021; Saravana Bhavan Venkatachalam et al., 2023). All PCR reactions were performed at a final volume of 25 μl, consisting of 5 μl of DNA template and 20 μl of reaction mixture. The reaction mixture contained 1.0 U HotStarTaq Plus DNA Polymerase (5 U/μl), 0.5 μl dNTP mix (10 mM), 0.5 μl of each primer (50 μM), 2.5 μl of 10× CoralLoad PCR buffer (including 15 mM MgCl_2_), and 15.8 μl of nuclease-free distilled water. For nested *Lankesterella* PCR assays, 2 μl of the first-round (outer) PCR product was used as template for the second-round amplification. The thermal cycling conditions applied for all four PCR assays are summarized in Table 1. Sequence-verified positive controls were used in each PCR run, including *Sarcocystis rileyi* (No. SR5), *Toxoplasma gondii* (No. TACH Tox1, WC18D), *Neospora caninum* (NC-1) and *Lankesterella* sp. (PB75 from this study, used after the initial detection of *Lankesterella* DNA).

**Table 1:** Primers and details for conventional PCR methods used in this study.

Target group	Target gene	Primer name	Primer sequence (5′-3′)	Amplicon size (bp)	Thermocycling profile	Reference
**Screening PCR**						
*Toxoplasma* spp., *Neospora* spp., *Sarcocystis* spp., *Isospora* spp.	18S rRNA	COC1*	AAGTATAAGCTTTTATACGGCT	∼350	94 °C for 10 min; 40× (94 °C for 30 s; 54 °C for 30 s; 72 °C for 30 s); 72 °C for 10 min	Ho et al. (1996)
		COC2*	CACTGCCACGGTAGTCCAATAC			
**Genotyping PCR**						
*Lankesterella* spp., *Toxoplasma gondii*	18S rRNA	Outer EF	GAAACTGCGAATGGCTCATT	∼1550	95 °C for 5 min; 35× (95 °C for 40 s; 55 °C for 1 min; 72 °C for 1.5 min); 72 °C for 7 min	Saravana Bhavan Venkatachalam et al. (2023)
		Outer ER	CTTGCGCCTACTAGGCATTC			
		Nested Hep153F	GTAATTCTATGGCTAATACATGCGC	∼1300	95 °C for 5 min; 35× (95 °C for 40 s; 57 °C for 1 min; 72 °C for 2 min); 72 °C for 7 min	
		Nested Hep1496R	TTATTGCCTCAAACTTCCTTGCG			
*Lankesteralla* spp., *Isospora* spp.	18S rRNA	Outer Cocc18S_n1F	CAGCTTTCGACGGTATGGTATTGG	∼1135	95 °C for 5 min; 35× (95 °C for 40 s; 58 °C for 1 min; 72 °C for 1.5 min); 72 °C for 7 min	Chagas et al. (2021)
		Outer Cocc18S_n1R	CAGACCTGTTATTGCCTCAAACTTCCT			
		Nested Cocc18S_n2F	GTATTGGCTTACCGTGGCAGTGAC	∼1105		
		Nested Cocc18S_n2R	GCCTCAAACTTCCTTGCGTTAGACA			

Purification and sequencing of PCR products were done by Eurofins Biomi Ltd. (Gödöllő, Hungary). PCR products from screening assays were sequenced in one direction, and longer amplicons from genotyping assays were sequenced bidirectionally. Only good-quality sequences were processed further. Obtained sequences were manually edited using the BioEdit program, then compared to GenBank sequences by the BLASTN program (https://blast.ncbi.nlm.nih.gov). The newly generated sequences were submitted to the GenBank database under the accession numbers PX925638-PX925640. All sequences retrieved from GenBank were aligned and subsequently trimmed to the length of the shortest sequence prior to phylogenetic analysis. This dataset was resampled 1000 times to generate bootstrap values. Phylogenetic analyses of *Lankesterella* and *Isospora* species were conducted with the Maximum-Likelihood and Neighbor-Joining methods, respectively, using the program MEGA 11 (Tamura et al., 2021). The best-fitting nucleotide substitution model was determined using IQ-TREE 2 version 2.4.0 (Minh et al., 2020).

### 2.3 Statistical analysis

Prevalences were compared with Fisher’s exact test (https://www.langsrud.com/fisher.htm), and differences were considered significant at *P* < 0.05.

## 3 Results

**Fig. 1:**
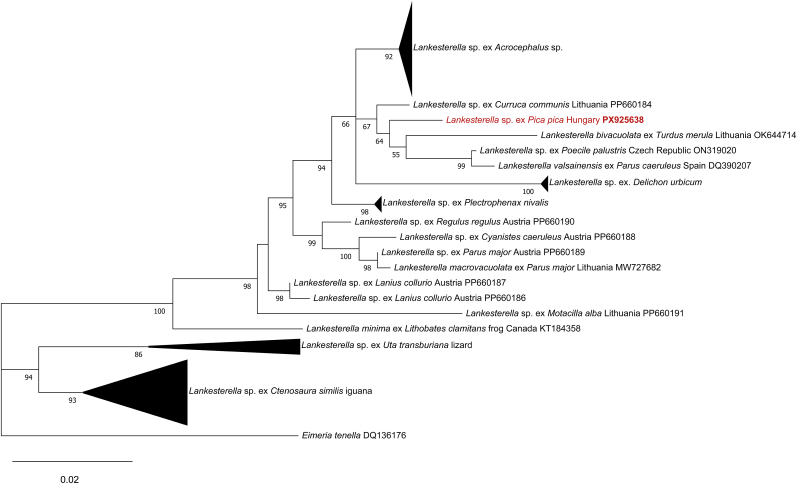
Phylogenetic tree of *Lankesterella* spp. based on the 18S rRNA gene. The sequence from this study is shown in bold. Sequence alignment was performed using the MUSCLE algorithm. The analysis included 54 nucleotide sequences, resulting in a final dataset comprising 1027 aligned positions. The best-fitting nucleotide substitution model was determined using IQ-TREE 2 v.2.4.0, and the selected model was TIM3+F+I+G4. Maximum-likelihood phylogenetic analysis was conducted in IQ-TREE 2 with 1000 ultrafast bootstrap replicates. *Eimeria tenella* (GenBank: DQ136176) was used as the outgroup. The resulting phylogenetic tree was visualized using MEGA 11.

In the screening assay, only the DNA samples from *P. pica* were PCR-positive, with a prevalence of 7% (19/273) (Table 2). PCR amplification and sequencing the nearly 1300 bp long fragment of the 18S rRNA gene showed that 11 samples (4%) contained the DNA of a *Lankesterella* sp., all representing the same genotype from both sampling locations (Table 2). The prevalence was 6.7% (4/60) in Budapest, and 3.3% (7/213) in Dömsöd, without a significant difference. The genotype (*Lankesterella* sp. from *Pica pica*, Hungary) showed the highest nucleotide sequence similarity (98.7%; 1021/1034 bp) to a *Lankesterella* genotype detected in the common whitethroat (*Curruca communis*) in Lithuania (GenBank: PP660184). The most genetically divergent *Lankesterella* isolate (GenBank: OP522457), reported from the marsh tit (*Poecile palustris*), shared only 96.3% sequence identity (902/937 bp) with the *P. pica*-derived genotype (Table 2). The phylogenetic analysis illustrates the genetic relatedness of this genotype to other *Lankesterella* lineages (Fig. 1).

**Table 2:** Results of molecular analyses from brain DNA extracts of Eurasian magpies.

Target category	*n/N* (Prevalence)	No. of detected 18S rRNA genotypes	GenBank ID	Minimum to maximum sequence identity percentages of the genotype from this study to GenBank sequences within the target category [GenBank IDs of reference sequences]
*Lankesterella* spp.	11/273 (4%)	1	PX925638	96.3–98.7% (902/937–1021/1034 bp) [OP522457 and PP660184]
*Toxoplasma gondii*	10/273 (3.7%)	1	PX925639	98.1–100% (1001/1020–1012/1012 bp) [X65508 and M97703]
*Isospora* spp.	1/273 (0.4%)	1	PX925640	98.2–99.6% (1010/1028–1037/1041 bp) [PP660174 and MW422271]

*Abbreviations*: *n*, number of sequence-verified positives; *N*, number of screened.

*Notes*: In the last column, minimum to maximum sequence identity ranges of the genotype from this study to GenBank sequences are relevant to the target category indicated in the first column (i.e. within the genera *Lankesterella* and *Isospora*, or within the species *Toxoplasma gondii*).

In addition, *T. gondii* DNA was detected in the brain tissue of 10 *P. pica* (3.7%), from both sampling locations (Table 3). The prevalence was 5% (3/60) in Budapest and 3.3% (7/213) in Dömsöd, without a significant difference. In four of these samples (PB72, PB79, PB91, and PB152), amplification of the long fragment of the 18S rRNA gene was successful. The obtained sequences showed 100% identity (1012/1012 bp) with reference sequences of *T. gondii* strain RH (GenBank: M97703) (Table 2) and other strains/isolates. On the other hand, it had only 98.1% (1001/1020 bp) sequence identity to the cyst-forming strain T626 (GenBank: X65508). In three samples (PB38, PB170, and PB231), mixed infections of *Toxoplasma* and *Lankesterella* were detected (Table 3).

**Table 3:** Results of sequencing from PCR-positive brain DNA extracts from *Pica pica*. Different sequencing results of the screening and genotyping PCRs (i.e. mixed infections) are indicated in bold. Sequencing was unsuccessful from the screening PCR for sample PB240, which showed weak positivity, and from the genotyping PCR in three cases.

Sample ID	Location	Result of sequencing	
		Screening PCR	Genotyping PCR
PB17	Dömsöd	*Lankesterella*	*Lankesterella*
PB38	BUD Airport	***Toxoplasma***	***Lankesterella***
PB72	BUD Airport	*Toxoplasma*	*Toxoplasma*
PB75	BUD Airport	*Lankesterella*	*Lankesterella*
PB79	BUD Airport	*Toxoplasma*	*Toxoplasma*
PB91	Dömsöd	*Toxoplasma*	*Toxoplasma*
PB97	Dömsöd	*Toxoplasma*	ns
PB121	Dömsöd	*Lankesterella*	*Lankesterella*
PB133	BUD Airport	*Lankesterella*	*Lankesterella*
PB138	BUD Airport	*Lankesterella*	*Lankesterella*
PB152	Dömsöd	*Toxoplasma*	*Toxoplasma*
PB170	Dömsöd	***Toxoplasma***	***Lankesterella***
PB215	Dömsöd	*Lankesterella*	*Lankesterella*
PB220	Dömsöd	*Toxoplasma*	ns
PB227	Dömsöd	*Lankesterella*	*Lankesterella*
PB231	Dömsöd	***Toxoplasma***	***Lankesterella***
PB240	Dömsöd	ns	*Isospora*
PB252	Dömsöd	*Lankesterella*	*Lankesterella*
PB255	Dömsöd	*Toxoplasma*	ns

*Abbreviation*: ns, not successful.

**Fig. 2:**
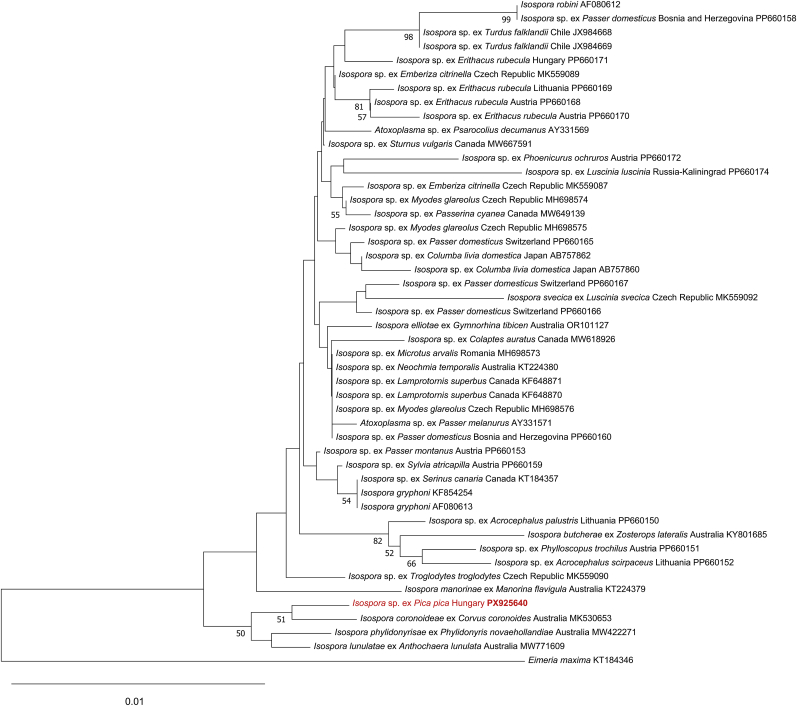
Phylogenetic tree of *Isospora* spp. based on the 18S rRNA gene. The sequence from this study is shown in bold. Evolutionary history was inferred using the Neighbor-Joining method, because the sequences were relatively similar to each other implying smaller evolutionary distances, in the case of which this method has high accuracy (Zou et al., 2024). The percentage of replicate trees in which the associated taxa clustered together in the bootstrap test (1000 replicates) is shown above the branches. The evolutionary distances were computed using the p-distance model. The tree is drawn to scale, with branch lengths measured in the number of substitutions per site. This analysis involved 48 nucleotide sequences. There were a total of 1030 positions in the final dataset. Evolutionary analyses were conducted in MEGA 11.

DNA of an additional apicomplexan species was detected in the brain tissue of one *P. pica* (PB240) from the Dömsöd area (Table 3). The obtained sequence exhibited the highest (99.6%; 1037/1041 bp) identity with *Isospora phylidonyrisae*, a coccidian species recently described from the New Holland honeyeater (*Phylidonyris novaehollandiae*) in Australia (GenBank: MW422271). Furthermore, the sequence showed 99.5% identity (1036/1041 bp) with *Isospora coronoideae* reported from the Australian raven (*Corvus coronoides*; GenBank: MK530653), a species belonging to the family Corvidae. However, the *Isospora* genotype detected in this study had only 98.2% (1010/1028 bp) sequence identity to an *Isospora* sp. reported from the thrush nightingale (*Luscinia luscinia*) in the northeastern-European exclave of Russia, Kaliningrad (GenBank: PP660174). The phylogenetic relationships of the Hungarian sequence are presented in Fig. 2. The clustering of the isolate from *P. pica* sampled in Hungary and the above isolates from Australia received only weak (50%) support.

In this study, no DNA of *N. caninum* and *Sarcocystis* spp. was detected. None of the samples from *G. glandarius* and *C. cornix* was PCR-positive; however, the sample sizes for these two species were very small.

## 4 Discussion

Eurasian magpies, *P. pica*, are increasingly urbanized, synanthropic birds occurring throughout the Palearctic (Jerzak, 2001), including Central Europe and Hungary (Faragó et al., 2017). Many corvid species are highly adaptable generalists that benefit from urbanization and often show increased occurrence in urban environments. Their advanced cognitive abilities, flexible nesting strategies, omnivorous diet, and capacity to exploit anthropogenic resources enable them to thrive under urban conditions (Abou Zeid et al., 2023), thereby creating opportunities for the transmission of various pathogens, including unicellular parasites, potentially even to companion animals kept outdoors.

In this study, 7% of the brain samples examined from *P. pica* tested PCR-positive for apicomplexan parasites. The brain was chosen as the target tissue of screening because of the neurotropism of *T. gondii* and *N. caninum*. However, while all apicomplexan parasites targeted here are known to be able to cross the blood-brain barrier, there can be differences in the infectious status of various tissues in passeriform birds, as for instance, *Lankesterella* spp. can be more frequently found in the brain than *Isospora* spp. (Keckeisen et al., 2024). Therefore, it is possible that the actual prevalence of infection was higher in *P. pica* of this study, depending on the group of apicomplexan parasites. Furthermore, based on sequencing results from three birds, mixed infections with *T. gondii* and *Lankesterella* were also detected.

Among the samples, 4% contained the DNA of *Lankesterella* species. A large-scale survey analyzing blood and tissue samples, including brain tissue, from 815 live wild birds and 15 deceased individuals reported an overall *Lankesterella* prevalence of 10.7% (Keckeisen et al., 2024), which is higher than that observed in our study. However, direct comparison is precluded, as our investigation involved a smaller sample size and focused exclusively on brain tissue from corvids. In the study by Keckeisen et al. (2024), samples were collected in Slovakia, Austria, Lithuania, Hungary, Switzerland, Bosnia and Herzegovina, and Russia, and were predominantly obtained from small passerines. Among these, European robins (*Erithacus rubecula*) and Eurasian blackcaps (*Sylvia atricapilla*) exhibited the highest infection rates of *Lankesterella* spp. Although Eurasian jays were also screened, no *Lankesterella* infections were detected (Keckeisen et al., 2024). Nevertheless, in other studies, *Lankesterella* spp. have also been identified in several passerine species, but not in *P. pica* (Merino et al., 2006; Biedrzycka et al., 2013; Chagas et al., 2021; Gutiérrez-Liberato et al., 2025; Saravana Bhavan Venkatachalam et al., 2025).

To the best of our knowledge, this is the first report of *Lankesterella* infection in *P. pica*, and in corvids in general. The isolate identified in *P. pica* in our study may represent a host-associated genotype, based on the presence of a single genetic variant in all infected individuals at two locations, as also supported by the sequence divergence from previously described *Lankesterella* taxa. However, because of the limited taxonomic resolution of 18S rRNA sequences in this genus, as also reflected by the low bootstrap support values in the phylogenetic analyses (Fig. 1), no ultimate conclusion can be drawn on the exact taxonomic status of the *Lankesterella* sp. identified in *P. pica* during this study.

Considering current knowledge on the evolutionary history and host associations of avian-infecting *Lankesterella* spp. (Chagas et al., 2021; Saravana Bhavan Venkatachalam et al., 2025), transmission is presumed to occur *via* orally ingested blood-feeding arthropod vectors. Although this raises the theoretical possibility that captive birds housed outdoors (e.g. in aviaries) could be exposed to infection through vectors that previously fed on infected *P. pica*, such spillover events are considered unlikely given the strong host specificity observed in *Lankesterella* spp. infecting other passerine families, including Paridae and Acrocephalidae (Saravana Bhavan Venkatachalam et al., 2025).

The present results also showed that 3.7% of the examined birds were infected with *T. gondii*. Birds play an intermediate host role in the life cycle of this parasite, and the prevalence of infection in cats may reflect infection levels in local avian and rodent communities, owing to their role as primary prey species (Hill and Dubey, 2002; Djurković-Djaković et al., 2019).

Nevertheless, there is limited research on the occurrence of *T. gondii* in corvids. In Europe, a survey conducted in Spain in 2011 examined brain samples from 200 wild birds using PCR, of which 12 (6%) tested positive for *T. gondii*; five of these were from the Eurasian jay (*G. glandarius*) and five from *P. pica* (Darwich et al., 2012). In the same year in Spain, *T. gondii* was detected in 80.5% of common ravens (*C. corax*) using a modified agglutination test; these birds, interestingly, exhibited aggressive behavior, including attacks on newborn animals and the consumption of dead fetuses (Molina-López et al., 2012). Furthermore, in Italy, myocardium samples collected in 2020 revealed *T. gondii* infection in 5.8% of the examined birds (41 *P. pica* and 4 hooded crows, *C. cornix*), as determined by modified agglutination test; however*, T. gondii* DNA was detected in only 15 of the 45 seropositive individuals (Mancianti et al., 2020). In addition, in Romania, *T. gondii* antibodies were detected at high prevalence in four corvid species: jackdaw (*C. monedula*) (22.6%); rook (*C. frugilegus*) (28.2%); hooded crow (*C. cornix*); and *P. pica* (12.5%). However, the DNA of *T. gondii* was identified by PCR in the heart muscle of only three individuals, of which one was *P. pica* (Gherman et al., 2025). On a global scale, *T. gondii* was detected in 9 out of 55 brain tissue samples collected from *C. cornix* in Iran, in 2018, corresponding to a prevalence of 16.3% (Abdoli et al., 2018). Compared with these reports, our study observed a lower infection rate in corvids, which may be attributable to the relatively low prevalence of *T. gondii* in Hungary, where the infection is reported to affect only approximately one third of the cat population and appears to be decreasing (Hornok et al., 2008; Dunay et al., 2024).

Notably, to the best of our knowledge, our study provides the first detection of *T. gondii* in *P. pica* in Hungary and in a broader geographical range of Central Europe. It has to be noted that sequencing a longer, 1012 bp long part of the 18S rRNA gene was only successful from four samples in our study, probably owing to low template quantity or DNA degradation. Based on the comparison of this longer 18S rRNA sequence with GenBank data, our isolate showed 100% identity to the highly virulent RH strain (genotype I) and the widely distributed ME49 strain (genotype II) (Fernández-Escobar et al., 2022; Zheng et al., 2025). However, in the context of *T. gondii*, the aim of molecular analysis was only the confirmation of the species based on a second molecular marker, because the 18S rRNA gene alone is often insufficient to molecularly characterize the strains/isolates of *T. gondii*, and its polymorphism does not correlate with virulence (Luton et al., 1995). This also explains why comparisons of 18S rRNA sequences derived from *P. pica* did not allow accurate determination of *T. gondii* strain in this study.

Since *P. pica* is known to consume dog and cat feces as part of its diet (Clarkson et al., 1986), it may become infected not only through the ingestion of tissues from other intermediate hosts but also directly from oocyst-containing feces of definitive host cats. According to our results, *Toxoplasma*-carrying *P. pica*, particularly those occurring in gardens, may serve as potential intermediate hosts and could be involved in the local maintenance of *T. gondii*. However, their exact role in the transmission to domestic animals such as dogs and cats remains unclear.

Based on sequence comparisons, the closest relatives of the *Isospora* sp. identified in one *P. pica* in Hungary are *I. phylidonyrisae* and *I. coronoideae*, which, to the best of our knowledge, have previously been reported exclusively from avian hosts in Australia (Liu et al., 2019a; Yang et al., 2021). Interestingly, isolates genetically similar to avian *Isospora* spp. have been identified in rodents from Central Europe (Fig. 2); however, these were regarded as pseudoparasites, as they presumably originated from avian fecal contamination (Trefancová et al., 2019). Thus, our study provides, to the best of our knowledge, the first direct evidence for the presence of relatives of these Australian *Isospora* spp. in a European avian host. It is worth mentioning that systemic isosporosis has been described only in passeriform birds (Flach et al., 2022), and brain involvement has been reported in only a few species, but not previously in corvids (Keckeisen et al., 2024). Furthermore, as the DNA was detected in the brain tissue of the affected *P. pica*, our findings also support a previously unconfirmed stage in the developmental pathway of passerine-infecting *Isospora* species, namely their dissemination from the intestinal wall to the central nervous system *via* lymphoid cells and/or macrophages (Keckeisen et al., 2024).

Based on the sequence comparisons of the *Isospora* genotype detected in *P. pica*, i.e. it had only 0.4–0.5% difference from Australian *Isospora* spp., this is the first indication that close relatives of the latter are also present in distant geographical locations. While in this context the low level of 18S rRNA sequence divergence may reflect genetic exchange between geographically distant conspecific isolates *via* bird migration or transportation (e.g. as pet birds), it has to be taken into account that avian *Isospora* spp. are known for their high degree of host specificity (Kubiski et al., 2022). Owing to the limited resolution of 18S rRNA sequences, further genetic markers will be needed to elucidate the taxonomic status of this species from *P. pica*.

The above findings provide novel data on the molecular detection of three apicomplexan lineages in brain tissue from *P. pica*, including first reports from a geographical point of view or at the host level. However, these findings should be interpreted in light of the study’s limitations, which include disproportionate sampling of different corvids, single-tissue analysis, lack of histopathology, single-locus molecular identification, absence of quantitative parasite load estimation, and the possibility that detected DNA does not reflect active infection.

Nevertheless, the results highlight the presence of DNA of these parasites in *P. pica* and support future epidemiological investigations. The simultaneous presence of these birds with domestic (pet and livestock) animals as well as humans in similar peri-urban locations, as studied here, deserves stronger ecological and One Health interpretations. These will require additional indicators of active protozoan infection, experimentally verified transmission routes and optimally the assessment of the epidemiological involvement of a broader spectrum of corvids.

## 5 Conclusions

Our findings broaden current knowledge of apicomplexan parasites infecting *P. pica* by documenting novel host and geographical occurrence and highlighting the diversity of these protozoans in peri-urban corvid populations. Further multidisciplinary studies are warranted to clarify their taxonomy, epidemiology, and ecological significance, including their potential role in parasite circulation at the wildlife-domestic animal-human interface.

## Ethical approval

All birds were culled for population control purposes in accordance with national regulations (Decree No. 79/2004 (V.4.) of the Ministry of Agriculture and Rural Development on the implementation of Act LV of 1996 on the protection of game, game management, and hunting).

## CRediT authorship contribution statement

**Barbara Tuska-Szalay:** Writing - original draft, Writing - review & editing. **Klaudia Gaál:** Investigation, Writing - review & editing. **Martin Horváth:** Investigation, Writing - review & editing. **Gergő Keve:** Visualisation, Writing - review & editing. **Lajos Szabó:** Investigation, Writing - review & editing. **Imre Gergely:** Investigation, Writing - review & editing. **Sándor Hornok:** Conceptualization, Methodology, Writing - original draft, Writing - review and editing.

## Funding

This study was funded by the 10.13039/100020916Hungarian Research Network (HUN-REN), Hungary (Project No. 1500107).

## Declaration of competing interests

The authors declare that they have no known competing financial interests or personal relationships that could have appeared to influence the work reported in this paper.

## Data Availability

The sequences obtained during this study are deposited in the GenBank database under the accession numbers PX925638-PX925640. All other relevant data are included in the article and its supplementary file.
